# Sexual Violence Against Mental Health Nurses in Inpatient Psychiatric Settings: A Systematic Review of Prevalence, Outcomes, and Risk Factors

**DOI:** 10.3390/nursrep16020059

**Published:** 2026-02-10

**Authors:** Giuliano Anastasi, Marika Lo Monaco, Mariachiara Figura, Daniela D’Amico, Emanuele Amodio, Alessandro Stievano, Ippolito Notarnicola, Roberto Latina

**Affiliations:** 1 Department of Medicine and Surgery, University of Enna “Kore”, 94100 Enna, Italy; giuliano.anastasi@unikore.it (G.A.); ippolito.notarnicola@unikore.it (I.N.); 2 Department of Health Promotion Sciences, Maternal and Infant Care, Internal Medicine and Medical Specialties (PROMISE), University of Palermo, 90133 Palermo, Italy; marika.lomonaco@unipa.it (M.L.M.); daniela.damico@community.unipa.it (D.D.); emanuele.amodio@unipa.it (E.A.); roberto.latina@unipa.it (R.L.); 3 Department of Clinical and Experimental Medicine, University of Messina, 98122 Messina, Italy; alessandro.stievano@unime.it

**Keywords:** sexual violence, workplace violence, mental health, psychiatry, prevalence, risk factors, nurses, healthcare workers, review

## Abstract

**Background/Objectives**: Workplace violence (WPV) is a major occupational concern in psychiatric settings, where mental health nurses (MHNs) are consistently identified as a high-risk professional group. Within this context, sexual violence (SV) remains understudied as a distinct phenomenon and is often embedded within aggregated measures of WPV. This systematic review aimed to synthesize the available evidence on SV against MHNs working in inpatient settings by: (1) describing its prevalence, forms, and characteristics; (2) examining psychological, occupational, and physical outcomes; and (3) identifying associated risk factors. **Methods**: This systematic review was conducted in accordance with PRISMA guidelines and registered in PROSPERO (CRD420251103606). A literature search was performed across PubMed, CINAHL, Scopus, Web of Science, and PsycInfo, supplemented by reference list checking and citation tracking. Peer-reviewed quantitative and qualitative studies published in English or Italian were eligible if they involved MHNs working in inpatient settings and addressed SV. Study selection, data extraction, and risk-of-bias assessment were conducted independently by two reviewers. A narrative synthesis following SWiM guidance was undertaken, and the certainty of evidence for statistically significant outcomes was assessed using the GRADE approach. **Results**: Twenty-five studies published between 2003 and 2025 were included. Definitions of SV varied substantially. Reported prevalence ranged from 0% to 68%, with verbal sexual harassment ranging from 19.5% to 53.4%, physical sexual harassment ranging from 14% to 42.9%, and sexual assault up to 18.6%. Evidence indicated associations between SV exposure and poorer quality of life, burnout, and days lost from work. The main risk factors included gender, age, education, work experience, employment type, acute psychiatric settings, night shifts, normalization of violence, and history of physical and sexual violence. **Conclusions**: SV against MHNs represents a relevant issue in psychiatric settings. Findings suggest significant psychological and occupational consequences. Standardized definitions and measurement, longitudinal research, and intervention studies are needed to inform effective prevention strategies and organizational responses.

## 1. Introduction

Workplace violence (WPV) against healthcare workers (HCWs) is widely recognized as a critical occupational and public health issue [1,2,3], with significant implications for workers’ safety, well-being, and quality of care [2,4,5]. Global estimates indicate that more than half of HCWs experience some form of WPV during their careers [2,6,7], and recent reviews report high rates of verbal (66.8%), physical (20.8%), and sexual violence (SV) (10.5%) within healthcare settings [8].

Mental health settings (MHSs) appear to be significantly affected by WPV [1,4,7,9], with prevalence estimates reaching up to 76% [10] and as many as 85% of HCWs reporting exposure to WPV in MHSs [11]. Among them, mental health nurses (MHNs) are consistently identified as one of the professional groups at greatest risk [1,10,12]. In MHSs, clinical care is inherently intertwined with risk management, and safety has long been recognized as a core objective [13]. Indeed, HCWs consistently identify personal safety as a prerequisite for effective clinical practice and professional well-being in MHSs [14,15].

Previous research has identified multiple patient-related risk factors for WPV in MHSs, including younger age [9,10], male gender [12], severity of mental illness [9,10,12,16], alcohol [9,12] or drug use [9,10], and history of aggressive behavior [9,10,12,16]. In addition, several contextual factors have been associated with an increased risk of WPV, such as involuntary admission [16,17], higher bed occupancy [10,16], and the presence of unqualified or temporary staff [10]. Exposure to WPV has been associated with a wide range of adverse outcomes among mental HCWs, including reduced physical health [18] and quality of life (QoL) [19], depressive symptoms [20], psychological distress [21], burnout [22], post-traumatic stress (PTSD) [23], and impaired work functioning [20].

Within this broader literature, SV represents a distinct and sensitive form of WPV that has received comparatively limited focused attention in MHSs [24]. According to the World Health Organization (WHO), SV is defined as ‘any sexual act, attempt to obtain a sexual act, or other act directed against a person’s sexuality using coercion, by any person regardless of their relationship to the victim, in any setting’ [25] and encompasses a continuum of behaviors, including verbal sexual harassment, sexual advances, sexual abuse, and rape [26]. However, in studies conducted in MHSs, SV is often assessed alongside physical and verbal aggression or subsumed within aggregated measures of WPV [9,20], potentially due to the absence of consistent definitions for these concepts [11,27]. As a result, prevalence estimates, contextual characteristics, outcomes, and risk factors specifically attributable to SV are often difficult to interpret.

Emerging evidence suggests that nurses are more exposed to SV than other HCWs [28,29], reporting adverse mental (44.6%), physical (30.19%), and emotional (61.26%) outcomes [30]. Prior research indicates that nurses’ exposure to SV is a significant predictor of depression, anxiety, stress [31], poorer QoL, and increased turnover intentions [32], and is also associated with reduced job satisfaction [33], PTSD [34], and burnout [35]. Qualitative studies further highlight the emotional and behavioral impact of SV, describing experiences of fear, unsafety, and powerlessness [36], alongside avoidance behaviors, including visiting patients in groups [37], changing shifts, and leaving the wards [38].

Although previous systematic reviews have synthesized evidence on WPV against MHNs [20,39,40,41] and on SV against HCWs and nurses [30,42,43], to the best of our knowledge, no systematic review has specifically focused on SV against MHNs. The absence of a targeted synthesis limits the ability to accurately characterize the scope and nature of SV in this professional group, to distinguish between different forms of SV, and to identify consistent patterns in prevalence, outcomes, and associated risk factors.

Therefore, this systematic review aims to synthesize the available evidence on SV against MHNs in inpatient MHSs. The specific objectives are to: (1) describe the prevalence, forms, and characteristics of SV; (2) examine the psychological, occupational, and physical outcomes associated with exposure to SV; and (3) identify sociodemographic, professional, contextual, and experiential factors associated with increased risk.

## 2. Materials and Methods

### 2.1. Study Design

This systematic review was performed according to the Preferred Reporting Items for Systematic Reviews and Meta-Analyses (PRISMA) statement [44,45]. In addition, to improve the scientific rigor of the study, the PRISMA Checklist was used [45] (see Table A1), and the research protocol was registered in the PROSPERO (International Prospective Register of Systematic Reviews) database (ID: CRD420251103606).

### 2.2. Eligibility Criteria

The eligibility criteria for this systematic review were defined a priori and aligned with the review scope and methodology [44,45]. Criteria were formulated through consensus among the research team and structured according to a population–exposure–outcome framework [46] to ensure methodological clarity and reproducibility.

Inclusion criteria comprised primary empirical research studies published in peer-reviewed journals, employing either quantitative or qualitative designs (e.g., observational, descriptive, case series, quasi-experimental, or randomized controlled studies). Eligible studies involved MHNs (including registered nurses, licensed nurses, auxiliary nurses, nursing aides, and nursing assistants) of all genders, aged ≥ 18 years, working in inpatient MHSs (e.g., psychiatric hospitals and acute psychiatric units). Studies conducted in any country and published in any year were considered. Only articles written in English or Italian were included. To be eligible, studies had to address SV against MHNs, including sexual harassment, sexual assault, rape, or related sexual offenses.

Exclusion criteria included non-empirical studies, secondary research, gray literature, and non-peer-reviewed publications. Studies focusing on physical violence, verbal violence, or bullying, mobbing, and stalking were excluded. Research involving nursing students, non-nursing professionals (e.g., psychiatrists, psychologists, social workers), or nursing staff working outside inpatient MHSs was excluded. Articles published in languages other than English or Italian were excluded due to translation resource limitations. No time restrictions were applied to maximize the completeness of the evidence base.

### 2.3. Information Sources

To identify potentially relevant records, a systematic literature search was conducted across five databases: PubMed, CINAHL, Scopus, Web of Science, and PsycInfo. The database search was complemented by additional strategies, including reference list checking and citation tracking (snowballing). The final search was performed on 19 July 2025.

### 2.4. Search Strategy

A comprehensive search strategy was developed and implemented across all selected databases, guided by the population–exposure–outcome framework [46]. The search focused on: MHNs (population), SV (exposure), and related outcomes and risk factors. Search strings combined controlled vocabulary terms and free-text keywords using Boolean operators and were adapted to each database’s indexing systems.

In line with recommendations [44,45], full search strategies for each database were documented for transparency and reproducibility and are available in Table A2.

### 2.5. Selection Process

The selection process comprised two phases: title and abstract screening, followed by full-text screening of potentially eligible studies. All records were exported into EndNote© 20.2.1 (Clarivate Analytics, Philadelphia, PA, USA) for duplicate removal and then uploaded to Rayyan© (Qatar Computing Research Institute, Doha, Qatar) to facilitate screening [47].

Screening was performed independently by two reviewers (GA & DD) in a double-blind manner. In the first phase, titles and abstracts were assessed against the predefined eligibility criteria. Records deemed potentially relevant by both reviewers proceeded to full-text screening. Full texts were retrieved via EndNote© and online research and were independently evaluated for inclusion. Disagreements at any stage were resolved through discussion or, when necessary, by consultation with a third reviewer (RL).

The study selection process was documented using a PRISMA flow diagram, reporting the number of records identified, screened, included, and excluded, along with the reasons for full-text exclusions, in line with recommendations [44,45].

### 2.6. Data Collection Process

A data extraction form, informed by Cochrane recommendations [48], was used to collect relevant information from included studies. The template and data extraction were managed using Microsoft Excel© for Microsoft 365 (Microsoft Corporation, Redmond, WA, USA) and EndNote© 20.2.1 (Clarivate Analytics, Philadelphia, PA, USA) to ensure traceability and accuracy.

Data extraction was performed independently by two reviewers (GA & DD) to minimize errors and subjective bias. Disagreements were resolved through discussion or, when necessary, by consultation with a third reviewer (RL).

### 2.7. Data Items

Data extracted from the included studies were classified into predefined categories to ensure comprehensive reporting and analysis, in line with recommendations [45,48].

Extracted information included bibliographic details (authors and year); study design; country; sample characteristics (sample size and professional role); and type of SV (e.g., sexual harassment or sexual assault). The main findings were also recorded, including prevalence estimates, identified consequences, and risk factors of SV, together with corresponding measures of effect where available.

### 2.8. Study Risk-of-Bias Assessment

In line with the PRISMA guidelines [44,45], the methodological quality and risk of bias of included studies were systematically assessed. The Joanna Briggs Institute (JBI) Critical Appraisal Tools [49] were used for cohort, qualitative, and cross-sectional studies, while the Mixed Methods Appraisal Tool [50] was applied to mixed-method studies.

Each checklist item was evaluated using the predefined options (“Yes”, “No”, “Unclear”, or “Not Applicable”), and total scores were calculated based on the number of ‘Yes’ responses. The maximum scores were 11 for cohort, 10 for qualitative, 9 for cross-sectional, and 7 for mixed-method studies. Risk of bias was classified as high (<50%), moderate (50–70%), or low (>70%) based on the proportion of ‘Yes’ responses.

Assessments were conducted independently by two reviewers (GA & DD), with disagreements resolved by a third reviewer (RL). Findings of the risk-of-bias assessment were considered in the evaluation of the overall certainty of the evidence.

### 2.9. Effect Measures

Effect measures included prevalence estimates of SV and measures of association such as crude rate ratio (CRR), odds ratios (ORs), and correlation coefficients, depending on the study design and analyses. Due to heterogeneity in designs, outcomes, and statistical methods, effect measures were reported as originally presented.

### 2.10. Synthesis Methods

Although a meta-analysis was initially considered, substantial heterogeneity across studies precluded pooled quantitative synthesis, in accordance with Cochrane recommendations [48]. Heterogeneity related to study designs, definitions, and forms of SV, outcome measures, and analytical approaches, limiting statistical comparability. A formal narrative synthesis was therefore conducted following the Synthesis Without Meta-analysis (SWiM) guidelines [51], in line with PRISMA standards [44,45].

For synthesis purposes, based on the review’s aims, studies were grouped a priori according to their focus into: (a) prevalence and characteristics of SV, (b) consequences of SV, and (c) risk factors for SV. For studies examining the prevalence and characteristics of SV, findings were reported narratively. For studies examining consequences of SV, findings were grouped into three conceptual categories: (1) psychological, (2) occupational, and (3) physical outcomes. For studies examining risk factors, findings were grouped into four conceptual categories: (1) sociodemographic, (2) professional, (3) contextual, and (4) experiential factors.

To summarize findings across heterogeneous effect measures, a vote-counting approach based on the direction of effect was applied. For the consequences of SV, the direction of effect was used to summarize the effect of exposure on outcomes (e.g., negative = worse outcome). For risk factors, the direction of effect was used to summarize the role of each factor in influencing the risk of exposure to SV (e.g., positive = higher risk; negative = lower risk; unclear = no clear association). When individual studies reported multiple consequences or risk factors, each relationship was considered separately.

Certainty of the evidence was assessed using the Grading of Recommendations Assessment, Development, and Evaluation (GRADE) approach [52]. This assessment was conducted independently by two reviewers (GA and MLM), with disagreements resolved by a third reviewer (IN). Evidence was rated as high, moderate, low, or very low, based on criteria related to risk of bias, inconsistency, indirectness, imprecision, and publication bias. As GRADE is designed for quantitative effect estimates [52], it was applied only to outcomes with statistically tested associations; however, qualitative and non-tested findings were considered in the narrative synthesis to triangulate evidence.

## 3. Results

### 3.1. Study Selection

**Figure 1:**
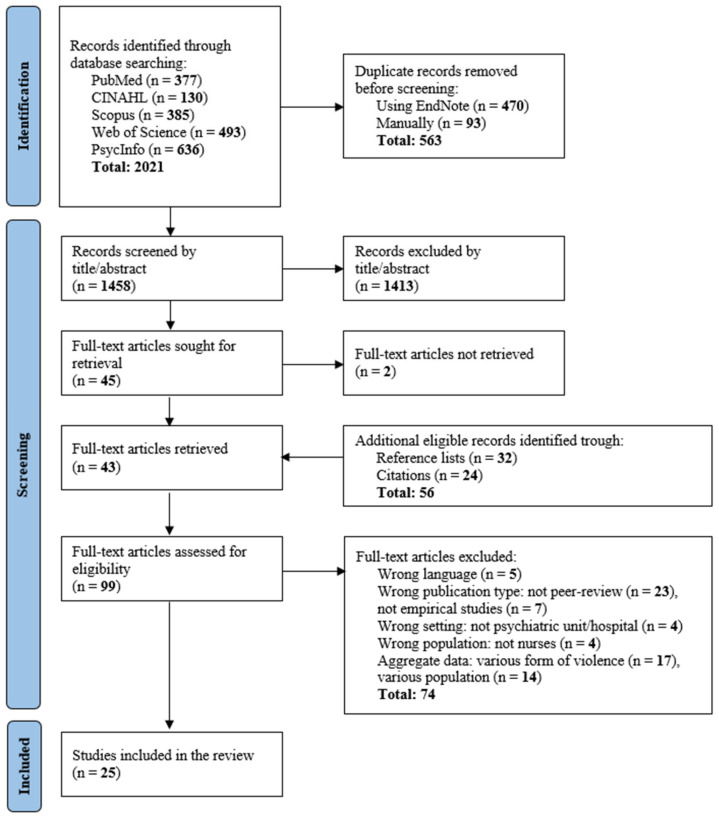
PRISMA flowchart showing the articles’ screening procedure, the number of sources included and excluded, and the reasons.

The database search identified 2021 records: 377 from PubMed, 130 from CINAHL, 385 from Scopus, 493 from Web of Science, and 636 from PsycInfo. After removal of 563 duplicates, 1458 records were screened by title and abstract, and 1413 records were excluded. Therefore, 45 full-text articles were sought, of which 43 were retrieved. An additional 56 records were identified through reference list checking (n = 32) and citation tracking (n = 24), resulting in 99 full-text articles assessed for eligibility. During full-text screening, 74 articles were excluded due to wrong language (n = 5), non-peer-reviewed publication (n = 23), non-empirical design (n = 7), wrong setting (n = 4), wrong population (n = 4), or use of aggregated data that precluded extraction of results specific to SV (n = 17) or MHNs (n = 14). Overall, 25 studies met the inclusion criteria and were included in the systematic review. The selection process and reasons for exclusion are detailed in the PRISMA flow diagram (Figure 1).

### 3.2. Study Characteristics

A total of 25 studies were included in this systematic review. The main characteristics of the included studies are summarized in Table 1, which provides an overview of publication year, study design, country, sample characteristics, form of SV investigated, and main findings.

Overall, the included studies were published between 2003 and 2025, showing that research on the topic spans more than two decades. Notably, more than one-third of the studies (36%; n = 9) [53,54,55,56,65,71,74,76,77] were published within the last five years.

Regarding study design, most of the included studies (72%, n = 18) [53,55,58,59,61,62,63,64,65,66,67,69,70,71,72,73,75,76] adopted quantitative cross-sectional designs, while a limited number employed qualitative (16%; n = 4) [54,56,74,77], cohort (8%; n = 2) [57,60], or mixed-method approaches (4%; n = 1) [68]. No experimental studies were identified.

The studies were conducted across a wide range of geographical contexts, including Asia [57,58,59,60,65,70,72,73,74,75,76], the Middle East [53,54,55,56,64], Europe [63,66,69,71], North America [61,68], Africa [62,77], and Oceania [67], revealing the global relevance of the phenomenon. In detail, nearly half of the included studies originated from Asia (44%; n = 11) [57,58,59,60,65,70,72,73,74,75,76], with Taiwan being the most represented country (24%; n = 6) [57,58,59,60,70,72].

Sample sizes varied considerably across studies, ranging from 9 [77] to 1449 [76] participants, with the majority of studies focusing exclusively on MHNs (84%; n = 21) [53,54,55,56,57,61,62,63,64,66,67,68,69,70,71,72,73,74,75,76,77], although some also included nursing aides or other nursing staff in the sample (16%; n = 4) [58,59,60,65]. Interestingly, some studies (12%; n = 3) [56,57,72] focused only on female MHNs.

The exposure of interest encompassed various forms of SV, including verbal sexual harassment, physical sexual harassment, and sexual assault. Part of the studies (36%; n = 9) [61,66,67,68,69,71,72,73,75] differentiated between verbal, physical, and assaultive forms (e.g., verbal sexual harassment and physical sexual harassment), while the majority (64%; n =16) [53,54,55,56,57,58,59,60,62,63,64,65,70,74,76,77] reported aggregated measures.

Concerning the main findings, 32% of the studies (n = 8) [54,55,56,57,59,63,64,70] reported only epidemiological data, including prevalence estimates and information about the characteristics of SV, such as the description of the event and its perpetrators. Consequences were examined in 36% of studies (n = 9) [53,58,68,69,71,73,74,76,77], with a small subset (16%; n = 4) [53,69,73,76] reporting statistically tested associations. Finally, 48% of studies (n = 12) investigated risk factors [60,61,62,65,66,67,69,71,72,73,75,76], identifying variables associated with a higher or lower risk of exposure to SV.

### 3.3. Synthesis of Findings

#### 3.3.1. Prevalence and Characteristics of Sexual Violence Against MHNs

Across the included studies, SV against MHNs emerged as a complex phenomenon, with variability in prevalence estimates and forms. As previously noted, SV was operationalized using diverse definitions. Consequently, prevalence estimates varied depending on the type of SV assessed.

When considering any form of SV, prevalence estimates ranged widely, from 0% to 68% across studies. Lower prevalence estimates were observed for attempted sexual assault (0%) [66], while higher prevalence estimates were reported for sexual harassment (68%) [69]. When disaggregated by type, verbal sexual harassment emerged as the most frequently reported form of SV, with prevalence estimates ranging from 19.5% [61] to 53.4% [73] and reaching up to 63.4% in studies combining verbal and physical harassment [73]. Physical sexual harassment was also commonly reported, with prevalence estimates ranging from 14% [71] to 42.9% [73]. In contrast, sexual assault was reported less frequently, with prevalence estimates ranging from 0% [66] to 18.6% [75].

Studies also provided insights into the characteristics, perpetrators, and contextual dynamics of SV. Among quantitative studies reporting perpetrator identity (24%; n = 6) [57,58,61,64,66,70], patients were identified as the primary source of SV, accounting for the majority of reported cases (76.2% to 100%) [57,58,64,66], followed by visitors (1.9–11.5%) [61,70] and staff members (1.4–9.5%) [58,61,70]. Qualitative studies further illustrated the breadth of sexually violent behaviors experienced by MHNs, including accusations of prostitution, being compelled to marry, hug, or kiss, unwanted touching of intimate body parts, grabbing of the face, choking, and attempted or completed forced sexual intercourse [56,74,77]. Qualitative evidence also highlighted responses adopted by MHNs following SV incidents, including leaving the location, physically distancing themselves from perpetrators, and adopting protective strategies such as maintaining physical distance and ensuring access to exit routes [74]. From an organizational perspective, reporting and follow-up procedures appeared inconsistent. Only a minority of incidents (38.1%) were investigated [58], and reported responses to perpetrators ranged from verbal warnings to no consequences [70]. Although some quantitative evidence suggested that SV incidents were reported to senior staff more frequently than other forms of WPV [59], qualitative and quantitative findings indicate that underreporting remains widespread. Barriers to reporting included feelings of shame and guilt, perceptions that reporting would be useless or unimportant, and fear of negative consequences [59,70]. Finally, perceptions regarding the preventability of SV varied. In some quantitative studies, a majority of exposed staff considered SV events preventable [58], whereas qualitative accounts described incidents as unexpected and unpredictable [74].

#### 3.3.2. Consequences of Sexual Violence Against MHNs

The consequences of SV against MHNs included psychological, occupational, and physical domains, indicating that exposure to SV may lead to adverse outcomes affecting MHNs’ well-being and professional functioning. Table 2 maps each included study to the corresponding outcome group and outcome, together with information on direction of effect, statistical testing, and study risk of bias.

Six studies (24%) reported psychological consequences [53,58,73,74,76,77]. Findings consistently indicated a negative direction of effect between exposure to SV and various psychological outcomes. Two studies reported a poorer QoL among MHNs exposed to sexual harassment or attempted rape [53,76]. One study reported higher emotional exhaustion following sexual harassment [73]. One study reported that 14.3% of MHNs exposed to sexual harassment suffered PTSD symptoms [58]. Two qualitative studies further supported a negative psychological impact of SV, describing emotional responses such as embarrassment, anxiety, fear, shock, discomfort, and feeling unsafe following SV [74,77].

Two studies (8%) reported occupational consequences [69,74]. Findings indicated a negative direction of effect between exposure to SV and occupational outcomes. One study reported an association between exposure to sexual harassment or sexual assault and days lost from work [69]. A qualitative study supported adverse occupational impacts, describing avoidance behavior and intention to leave the job following SV [74].

Two studies (8%) reported physical consequences [68,71]. Findings indicated a negative direction of effect between exposure to SV and physical outcomes. Both studies reported that exposure to SV was associated with the need for medical treatment following the incidents [68,71].

#### 3.3.3. Risk Factors of Sexual Violence Against MHNs

Several sociodemographic, professional, contextual, and experiential risk factors were identified as influencing exposure to SV. Table 3 maps each included study to the corresponding risk factor group and risk factor, together with information on direction of effect, statistical testing, and study risk of bias.

Six studies (24%) investigated the role of sociodemographic factors [60,66,69,71,73,75]. Gender showed heterogeneous directions of effect across studies and forms of SV. Two studies indicated a higher risk of sexual harassment among female MHNs [60,69], whereas two studies reported a higher risk of sexual harassment [73] and sexual assault among male MHNs [75]. In contrast, one study reported a lower risk of verbal and physical SV among male MHNs [71], and another study found no clear association between gender and sexual harassment [75]. Age showed a positive direction of effect in two studies, with a higher risk of sexual harassment reported among younger MHNs (<40 years) [69] and among middle-aged MHNs (40–44 years) [66]. Marital status showed no clear association with sexual harassment in one study [73]. Education demonstrated mixed directions of effect, with one study reporting a higher risk of sexual harassment among MHNs with a college education [60] and another study reporting no clear association between education level and sexual harassment [73].

Nine studies (36%) explored the role of professional factors [60,61,62,65,67,71,72,73,76]. Across these studies, work setting and ward type emerged as among the most consistently identified professional risk factors. Three studies showed a higher risk of sexual harassment [61,67,72] and sexual assault [61] among nurses employed in psychiatric settings compared to other settings (e.g., surgery). However, one study reported no clear associations between work settings and sexual assault [72]. Similarly, two studies reported a higher risk of sexual harassment among MHNs working in acute or intensive psychiatric wards [60,73], although one study reported no clear association between ward type and risk of SV [65]. Work experience also showed mixed patterns: three studies indicated a higher risk of sexual harassment among MHNs with shorter work experience [76] or fewer than four years of experience [60,71], whereas one study reported a lower risk of sexual harassment among MHNs with fewer than ten years of experience [62], and another study found no clear association between work experience and physical SV [71]. Two studies observed no clear associations for professional title (e.g., nurses or nursing assistants) [65,73] or employment type (e.g., permanent or temporary) [73] and risk of sexual harassment and SV. Work schedule also showed mixed findings, with one study reporting part-time work associated with a lower risk of physical SV, but no clear association for verbal SV [71]. Working shifts showed a positive direction of effect, with one study reporting a higher risk of sexual harassment among MHNs working night shifts [73].

Only one study (4%) focused on the role of contextual factors [73]. In this study, work activities and patient gender showed a positive direction of effects, with a higher risk of sexual harassment reported during routine nursing activities (e.g., administering therapy) and among MHNs caring for male patients [73]. In contrast, no clear associations were reported for the location of events, patient age, or patient residence [73].

Three studies (12%) examined the role of experiential factors [60,71,73]. A history of SV showed a positive direction of effects in one study, with a higher risk of both verbal and physical SV [71], while a history of physical violence was associated with a higher risk of verbal SV, but not physical SV [71]. Being worried about violence showed a positive direction of effect, with a higher risk of sexual harassment reported in one study [60]. Similarly, perceiving violence as part of the job (normalization of violence) was associated with a higher risk of verbal SV, but not physical SV, in one study [71]. Perceptions of preventability showed no clear association with sexual harassment [73].

Overall, evidence suggests that working in psychiatric and acute ward settings, shorter work experience, caring for male patients, prior experiences of violence, worry about violence, and normalization of violence are associated with a higher risk of SV against MHNs. However, several variables, particularly gender, education, and work experience, showed mixed or inconsistent directions of effect across studies and forms of SV, while others demonstrated no clear associations.

### 3.4. Risk of Bias

The results of the methodological quality assessment are reported in Table 4. Using the established classification criteria, most of the included studies (60%; n = 15) [53,54,55,56,58,63,65,70,71,72,73,74,75,76,77] were assessed as having a low risk of bias, while a smaller proportion (40%; n = 10) [57,59,60,61,62,64,66,67,68,69] were classified as having a moderate risk of bias. No study was classified as having a high risk of bias.

### 3.5. Quality of Evidence

The results of the quality of evidence assessment are reported in Table 5. The quality of evidence for the association between sexual harassment and QoL was rated as low, based on two cross-sectional studies [53,76]. Evidence supporting the association between sexual harassment and burnout was rated as low, as it relied on one cross-sectional study [73]. The quality of evidence for associations between sexual harassment and sexual assault with days lost from work was downgraded to very low due to reliance on one study [69] with a small sample size, use of unstructured measures, and moderate risk of bias.

## 4. Discussion

This systematic review synthesized evidence on SV against MHNs in inpatient MHSs, focusing on prevalence, characteristics, consequences, and risk factors. Overall, SV emerged as a relevant occupational issue for MHNs, with consistent evidence of adverse consequences and a heterogeneous pattern of associated risk factors.

A major finding is the wide variability in reported prevalence, likely reflecting the inconsistent operationalization and definitions of SV in the literature [27,78]. Despite this heterogeneity, a consistent pattern emerged: verbal sexual harassment was the most commonly reported form of SV [73], while physical assault was less frequently reported [61,66]. This gradient is consistent with the literature, which indicates that verbal forms of SV [42,79] and non-physical forms of WPV occur more frequently than physical ones, both in general nursing populations [30,41,80,81,82] and HCWs [1,7,83].

Concerning the nature of SV experienced by MHNs, the review highlighted a spectrum of behaviors, ranging from verbal harassment and sexualized insults to unwanted physical contact, coercive acts, and attempted or completed sexual assault [56,74,77], consistent with prior research on SV in healthcare settings [30,42]. Across the included studies, patients were frequently identified as the main perpetrators [57,64,66]; however, several studies reported SV perpetrated by visitors or staff [58,61,70], aligning with previous research [30,33,84]. This broader perpetrator profile challenges narratives that normalize violence as an inevitable part of mental health nursing [85,86], which risks minimizing harm, obscuring organizational accountability, and perpetuating tolerance of abusive behaviors [87].

The review provides converging evidence that SV is associated with adverse outcomes among MHNs. Exposure to SV showed a consistent negative direction of effect for psychological outcomes, including QoL, burnout, and PTSD [53,58,73,76]. Although the quality of evidence is low, consistency with qualitative narratives strengthens confidence in the overall pattern. Qualitative studies further described fear, shame, anger, helplessness, and persistent feelings of unsafety following SV incidents [56,74,77], consistent with research in other nursing populations linking SV to acute stress responses and long-term psychological sequelae [30,88,89]. These findings reinforce that SV should be conceptualized not only in terms of physical injury but also as a relevant psychological stressor with potential long-term implications for MHNs’ mental health and well-being [90]. Occupational outcomes such as absenteeism [69] and intention to leave the job [74] were less consistent but emerged as relevant concerns. These findings mirror previous research linking SV and WPV to reduced job satisfaction and workforce attrition [33,91,92]. In the context of ongoing staffing shortages [93] and high turnover rates [94] in MHSs, the occupational impact of SV may have system-level implications, potentially compromising continuity of care, team functioning, and patient safety. Although less frequently reported, physical consequences were also documented, including injuries requiring medical treatment [68,71]. The relative scarcity of physical harm reports likely reflects the predominance of non-physical SV and potential underreporting, as suggested by the literature [84,95,96], rather than limited severity. However, the interpretation of these consequences should consider study quality and risk of bias. Importantly, the negative direction of effect for psychological outcomes was observed in studies rated as having a low risk of bias, which strengthens confidence in the presence of an adverse association. In contrast, evidence for occupational and physical outcomes was derived from studies with a low-to-moderate risk of bias. These factors contributed to the low-to-very low GRADE ratings about the certainty of evidence and indicate that conclusions regarding occupational and physical consequences should be interpreted with caution.

Risk factors reveal a multilevel vulnerability shaped by sociodemographic, professional, contextual, and experiential factors. Gender emerged as a central but complex determinant, with some studies reporting higher exposure among female MHNs [60,69] and others among male MHNs [73,75]. This heterogeneity likely reflects gendered norms influencing both disclosure and reporting of SV [97,98]. For instance, previous research suggests that male nurses may underreport SV due to stigma, difficulties in being recognized as a victim, and concerns about credibility [99,100]. Rather than necessarily indicating contradictory patterns, these findings suggest that SV affects MHNs of all genders, through mechanisms shaped by power relations, social norms, and role expectations within MHSs [101]. Age seems to be a relevant risk factor, with younger MHNs showing increased exposure [66,69] according to the literature [43,79,96]. Also, professional experience was one of the most consistent risk factors, with shorter tenure associated with higher exposure [59,62,71]. Limited clinical experience may reduce situational authority, confidence in boundary setting, and familiarity with de-escalation strategies, increasing vulnerability [40]. Part-time work appeared to confer some protection [71], likely reflecting reduced exposure time rather than intrinsic differences. Contextual factors also shaped exposure. Working in psychiatry [61,67,72], acute units [60,73], as well as night shifts [73] was associated with a higher risk. These settings are characterized by higher patient acuity, reduced supervision, and increased isolation [102,103], which may amplify vulnerability to SV. Finally, experiential factors further contributed to risk. A history of sexual assault or physical attack increased the likelihood of subsequent exposure [71], supporting a cumulative risk model in which prior victimization heightens vulnerability to future violence [104]. Interpretation of risk factors should also consider study quality. Evidence for several risk factors (e.g., male gender, shorter work experience, part-time work, employment in psychiatric settings and acute ward, and prior experiences of violence) was derived from studies rated as having a low risk of bias, strengthening confidence in these associations. In contrast, other findings, particularly those related to female gender, younger age, and level of education, were supported by studies with a moderate risk of bias, which may partially explain the heterogeneous directions of effect observed. This pattern suggests that inconsistencies in sociodemographic risk factors may reflect both contextual differences and methodological limitations.

### 4.1. Implications for Practice and Policy

This review highlights the need to conceptualize SV as a preventable occupational issue rather than an inevitable aspect of mental health nursing. At the clinical level, SV should be systematically addressed within risk assessment, staff training, and safety planning, with an emphasis on the early identification of sexually inappropriate behaviors, boundary setting, and prevention strategies [33,43]. At the organizational level, clear policies defining SV, accessible and non-punitive reporting systems, and leadership commitment to zero tolerance are essential. Targeted attention to high-risk contexts, such as night shifts and acute wards, is warranted through structural interventions, including adequate staffing, environmental modifications, and supervision. At the policy level, standardized definitions and the employment of modern systems for violent event detection in MHSs [105] would improve comparability across institutions and support evidence-informed prevention strategies. Explicit integration of SV into WPV frameworks and occupational health policies may enhance recognition, accountability, and resource allocation [79].

### 4.2. Strengths, Limitations, and Future Research

To the best of our knowledge, this is the first systematic review to specifically examine SV against MHNs in inpatient MHSs. It offers an overview of the prevalence, characteristics, outcomes, and risk factors. The broad conceptualization of SV, including verbal and physical forms, reflects the heterogeneous nature of the phenomenon and reduces the risk of underestimation due to narrower definitions. Moreover, the exclusive focus on MHNs enhances the relevance of the findings, while systematic methods and structured quality appraisal strengthen the rigor.

Several limitations should nonetheless be acknowledged. First, substantial heterogeneity in SV definitions and measurement instruments limited comparability and precluded meta-analysis; therefore, findings should be interpreted as indicative patterns rather than pooled estimates. Second, the evidence base was largely composed of cross-sectional, self-report studies, limiting causal inference and increasing vulnerability to reporting biases. Given the sensitive nature of SV and the normalization of violence in MHSs, both prevalence and impact may be underestimated. Third, the uneven geographic distribution of evidence limits the generalizability of the findings. Fourth, relatively few studies formally tested statistical associations, and longitudinal evidence remains scarce, constraining conclusions regarding temporal relationships and cumulative effects.

Future research should prioritize longitudinal and mixed-method designs to clarify causal pathways and the evolution of psychological and occupational outcomes following SV. Qualitative studies are needed to understand organizational culture, leadership, reporting climates, and power dynamics shaping exposure and responses to SV. Greater standardization in the definition and measurement of SV is essential to improve comparability [106]. Finally, intervention research evaluating organizational, educational, and policy strategies is critically needed to inform clinical guidelines, occupational health policies, and the development of safer MHSs [30].

## 5. Conclusions

This systematic review provides the first synthesis of evidence on SV against MHNs working in inpatient MHSs. SV emerged as a relevant occupational concern, marked by variability in prevalence estimates and heterogeneity in definitions. Verbal sexual harassment was the most frequently reported form of SV, whereas sexual assault was less common. Qualitative evidence indicates that SV often occurs unexpectedly during routine care interactions and may lead to persistent fear, hypervigilance, and a sustained sense of unsafety. Exposure to SV was associated with adverse outcomes, including reduced QoL, burnout, PTSD symptoms, absenteeism, and in some cases, physical injury requiring medical treatment. Identified risk factors included sociodemographic (e.g., gender and age), professional (e.g., shorter work experience), contextual (e.g., acute wards and night shifts), and experiential factors (e.g., history of violence).

Overall, these findings underscore that SV against MHNs should not be regarded as a marginal or inevitable aspect of mental health nursing, but as a preventable threat to workforce well-being, staff retention, and service continuity. Clinical and organizational responses should explicitly address the full spectrum of SV through safety cultures, adequate staffing and supervision in high-risk contexts, and accessible, trusted, and non-punitive reporting systems supported by leadership accountability. Advancing the evidence base requires standardized definitions and measurement tools, longitudinal research to clarify causal pathways and trajectories, and interventions at organizational and policy levels. Addressing these priorities is essential to inform practice guidelines, occupational health policies, and the development of safer psychiatric care environments for MHNs.

## Figures and Tables

**Table 1:** Characteristics of the included studies.

Reference	Design	Country	Sample	Form of Sexual Violence	Main Findings
Abu El-Kass et al., 2025 [53]	Cross-sectional	Saudi Arabia	171 MHNs	SA (attempted) or SH	Prevalence: SA (attempted) or SH = 0.6% Consequences: associations between SA (attempted) or SH and poor QoL (*p* < 0.05).
Abu Khait et al., 2022 [54]	Qualitative descriptive	Jordan	27 MHNs	SV	Prevalence: SV = 3.7%.
Alenezi, 2024 [55]	Cross-sectional	Saudi Arabia	361 MHNs	SV	Prevalence: SV = 9.1%.
Alyousef & Alhamidi, 2022 [56]	Qualitative explorative	Saudi Arabia	16 MNHs (female)	SH	Characteristics: SH was perpetrated by both genders, in forms such as the inducement of sexual intercourse, being accused of prostitution, nudity, being forced to hug, being forced to kiss, touching of private body parts, choking, and forced sexual intercourse.
Chen et al., 2011 [57]	Cohort study	Taiwan	74 MNHs (female)	SH	Prevalence: SH = 1%. Characteristics: perpetrators were patients (100%).
Chen et al., 2008 [58]	Cross-sectional	Taiwan	69 MHNs, 131 NAs, 22 clerks	SH	Prevalence: SH = 9.5%. Characteristics: perpetrators were patients (76.2%) and staff (9.5%). In 38.1% cases, incidents were investigated. Exposed staff (71.4%) considered SH preventable. Consequences: 14.3% of exposed staff reported PTSD symptoms.
Chen et al., 2009 [59]	Cross-sectional	Taiwan	69 MHNs, 131 NAs, 22 clerks	SH	Prevalence: SH = 9.5%. Characteristics: staff exposed to SH reported the incident to senior staff more (*p* < 0.01) than staff exposed to other forms of violence. Exposed staff did not report the SH due to shame, guilt, and the consideration that reporting was useless.
Chen et al., 2009 [60]	Cohort study	Taiwan	167 (MHNs and NAs)	SH	Prevalence: SH = 4.4%. Risk factors: female gender (CRR 24.78), college education (CRR 24.78), shorter work experience (< 4 years, CRR 1.37), acute ward (CRR 52.98), and being moderately worried about violence (CRR 9.68).
Hesketh et al., 2003 [61]	Cross-sectional	Canada	276 MHNs	SH (verbal) SA	Prevalence: SH (verbal) = 19.5%; SA = 0.8%. Characteristics: perpetrators were patients (92.3%), visitors, and staff (1.9% each), with a higher percentage compared to other nursing specialties. Risk factors: MHNs were more likely to report incidents of verbal SH (42.9%) and SA (50%) compared to nurses working in other settings.
James et al., 2011 [62]	Cross-sectional	Nigeria	73 MHNs	SH	Prevalence: SH = 32.9%. Risk factors: MHNs with <10 years of work experience were more likely to report fewer incidents of SH (t = −2.40, df = 71, *p* < 0.02).
Jonker et al., 2008 [63]	Cross-sectional	The Netherlands	85 MHNs	SH	Prevalence: SH = 20%.
Khoshknab et al., 2012 [64]	Cross-sectional	Iran	183 MHNs	SV	Prevalence: SV = 5.5%. Characteristics: perpetrators were patients (100%).
Kobayashi et al., 2020 [65]	Cross-sectional	Japan	599 (398 MHNs, 195 NAs, 6 missing)	SV	Prevalence: SV = 2.6%. Risk factors: SV prevalence did not differ by occupation (MHNs vs. NAs) or ward (acute vs. other).
Maguire & Ryan, 2007 [66]	Cross-sectional	Ireland	87 MHNs	SH SA	Prevalence: SH = 18%; SA = 0%. Characteristics: perpetrators were patients (100%). Risk factors: MHNs of middle age (40–44 years) were more likely to report SH compared to older and younger colleagues (*p* = 0.04).
McKenna et al., 2003 [67]	Cross-sectional	New Zealand	68 MHNs	SH (verbal) SH (physical)	Prevalence: SH (verbal) = 51%; SH (physical) = 21%. Risk factors: MHNs were more likely to report verbal SH (*p* < 0.001) and physical SH (*p* = 0.08) compared to nurses working in other settings.
Moylan and Cullian, 2011 [68]	Mixed method	United States of America	110 MHNs	SA	Prevalence: SA = 2.7%. Characteristics: exposed MHNs were female (100%). Consequences: one exposed MHN reported that after examination in the emergency room, she was sent back to work on the same unit, and if she refused to return, she would be disciplined.
Nijman et al., 2005 [69]	Cross-sectional	The United Kingdom	154 MHNs	SH SA	Prevalence: SH = 68%, SA = 3%. Consequences: associations between SH and days lost from work (r = 0.38; *p* < 0.001); SA and days lost from work (r = 0.20; *p* = 0.014). Risk factors: female (*p* < 0.05) and young (<40 years, *p* < 0.05) MHNs were more likely to report SH.
Niu et al., 2019 [70]	Cross-sectional	Taiwan	429 MHNs	SH	Prevalence: SH = 32.4%. Characteristics: perpetrators were patients (97.1%), visitors (11.5%), and staff (1.4%). Perpetrators were verbally warned (62.6%) or had no consequences (12.2%). Exposed MHNs did not report SH because they considered reporting useless (46.8%) or not important (28.8%) or were afraid of consequences (14.4%).
Schlup et al., 2021 [71]	Cross-sectional	Switzerland	1128 MHNs	SV (verbal) SV (physical) SA	Prevalence: SV (verbal) = 39%; SV (physical) = 14%; SA = 5%. Consequences: exposed MHNs reported serious injuries requiring medical treatment. Risk factors: male MHNs had a lower risk of exposure to verbal SV (OR 0.49; *p* ≤ 0.001) and physical SV (OR 0.66; *p* = 0.08) compared to female MHNs. MHNs with less work experience (≤ 3 years) had a higher risk of exposure to verbal SV (OR 3.60; *p* ≤ 0.001) compared to more experienced (> 20 years) MHNs. No differences were found between different levels of experience and physical SV. MHNs working part-time had a lower risk of exposure to physical SV (OR 0.53; *p* = 0.05) compared to MHNs working full-time. No differences were found between employment time and verbal SV. A history of SA was associated with a higher risk of verbal (OR 4.04; *p* ≤ 0.001) and physical SV (OR 4.53; *p* ≤ 0.001). A history of physical violence was associated with a higher risk of verbal SV (OR 1.72; *p* < 0.01), but not physical SV. MHNs perceiving that violence is part of the job reported a higher risk of exposure to verbal SV (OR 1.12; *p* ≤ 0.001), but not physical SV.
Shiao et al., 2010 [72]	Cross-sectional	Taiwan	467 MHNs (female)	SH (verbal) SH (physical) SA	Prevalence: SH (verbal) = 47.5%; SH (physical) = 17.6%; SA = 3.9%. Risk factors: MHNs had a higher annual incidence of verbal SH (*p* = 0.005) and physical SH (*p* = 0.005), but not SA (*p* = 0.50), compared to nurses working in other settings.
Yang et al., 2018 [73]	Cross-sectional	China	245 MHNs	SH (verbal) SH (physical)	Prevalence: SH (verbal) = 53.4%; SH (physical) = 42.9%; SH (verbal and physical) = 63.4%. Characteristics: SH was correlated with physical attack (r = 0.549; *p* ≤ 0.001). Consequences: the annual frequency of SH significantly correlated with burnout dimensions: emotional exhaustion (r = 0.253; *p* ≤ 0.001) and depersonalization (r = 0.179; *p* ≤ 0.001). MHNs reporting at least one SH incident had a higher mean emotional exhaustion compared to those who did not report SH incidents (2.18 ± 1.26 vs. 1.65 ± 1.22; *p* = 0.003). Risk factors: male MHNs reported a higher mean incidence of SH compared to females (1.61 ± 1.34 vs. 0.93 ± 1.24; *p* ≤ 0.001). MHNs working in psychiatric intensive care units for adult males reported a higher mean incidence of SH compared to MHNs working in other settings (2.26 ± 1.44; *p* ≤ 0.001). MHNs working on night shifts reported a higher mean incidence of SH compared to MHNs working on day shifts (1.46 ± 1.38 vs. 1.01 ± 1.24; *p* = 0.013). MHNs reported a higher mean incidence of SH when providing routine treatment (e.g., administering medications) compared to when performing other activities (1.70 ± 1.54; *p* = 0.016). MHNs reported a higher mean incidence of SH perpetrated by male patients compared to SH perpetrated by females (1.60 ± 1.34; *p* ≤ 0.001). No differences were found between MHNs’ marital status, education, professional title, employment type, perceptions of preventability, locations of events, perpetrator’s age, perpetrator’s residence, and SH.
Yosep et al., 2023 [74]	Qualitative descriptive	Indonesia	40 MHNs	SH	Characteristics: SH mostly involved male patients and female MHNs, in forms such as asking for sexual intercourse, accusing of being a whore, compelling to get married, touching, grabbing the face, hugging from behind, forcibly embracing, being naked, forcibly kissing, and touching breasts and buttocks. Responses to SH included leaving the location of incidents and slapping/hitting the patients. Strategies to avoid SH were being careful, keeping distance from the patient, ensuring to have an exit, and maintaining a dominant position. SH was described as unexpected and unpredictable. Consequences: avoidance behavior (e.g., avoiding the patient and avoiding wearing makeup), discomfort, shock, fear, anxiety, and intention to leave the job.
Zeng et al., 2013 [75]	Cross-sectional	China	387 MHNs	SH (verbal) SH (physical) SA	Prevalence: SH (verbal) = 24.8%; SH (physical) = 16.0%; SA = 18.6%. Risk factors: male MHNs were more likely to experience SA (28.9% vs. 15.5%; *p* = 0.004) compared to female MHNs. No differences were found between genders and verbal or physical SH.
Zeng et al., 2020 [76]	Cross-sectional	China	1449 MHNs	SH	Prevalence: SH = 21.5% (exposure to incident: once = 8.4%; twice = 7.1%; three or more = 6.0%). Consequences: MHNs exposed to SH reported lower QoL scores for each domain (physical, *p* < 0.001; psychological, *p* < 0.001; social, *p* = 0.003; environmental, *p* = 0.001), compared to MHNs not exposed. Risk factors: MHNs with shorter work experience were more likely to experience SH (*p* = 0.02).
Zwane et al., 2022 [77]	Qualitative descriptive	Eswatini	9 MHNs	SH	Characteristics: female MHNs reported that caring for male patients exposes them to SH, especially on night shifts when only one MHN (themselves) is present. SH was perpetrated by both genders, in forms such as calling them wives, asking for sexual intercourse, being naked in front of them, touching buttocks, and attempting to rape. Consequences: embarrassment, feeling unsafe, and fear.

Legend: CRR = crude rate ratio, MHNs = mental health nurses, OR = odds ratio, PTSD = post-traumatic stress disorder, QoL = quality of life, SA = sexual assault, NAs = nursing assistants, SH = sexual harassment, SV = sexual violence.

**Table 2:** Summary of consequences of sexual violence against MHNs.

Reference	Form of Sexual Violence	Outcome Group	Outcome (Variable)	Direction of Effect	Risk of Bias	Statistical Testing
Abu El-Kass et al., 2025 [53]	SA (attempted) or SH	Psychological	QoL	Negative	Low	Yes
Chen et al., 2008 [58]	SH	Psychological	PTSD (emotional exhaustion)	Negative	Low	No
Moylan and Cullian, 2011 [68]	SA	Physical	Need for medical treatment	Negative	Moderate	No
Nijman et al., 2005 [69]	SH SA	Occupational	Day lost from work	Negative	Moderate	Yes
Schlup et al., 2021 [71]	SV (verbal) SV (physical) SA	Physical	Need for medical treatment	Negative	Low	No
Yang et al. 2018 [73]	SH (verbal) SH (physical)	Psychological	Burnout	Negative	Low	Yes
Yosep et al., 2023 [74]	SH	Psychological	Adverse emotional responses	Negative	Low	No
		Occupational	Avoidance behaviors Intention to leave the job	Negative		
Zeng et al., 2020 [76]	SH	Psychological	QoL	Negative	Low	Yes
Zwane et al., 2022 [77]	SH	Psychological	Adverse emotional responses	Negative	Low	No

Legend: QoL = quality of life; PTSD = post-traumatic stress disorder; SA = sexual assault; SH = sexual harassment; SV = sexual violence.

**Table 3:** Summary of risk factors for sexual violence against MHNs.

Reference	Form of Sexual Violence	Risk Factor Group	Risk Factor (Variable)	Direction of Effect	Risk of Bias	Statistical Testing
Chen et al., 2009 [60]	SH	Sociodemographic	Gender (female) Education (college)	Positive	Moderate	Yes
		Professional	Ward type (acute) Work experience (<4 years)	Positive		
		Experiential	Being worried about violence	Positive		
Hesketh et al., 2003 [61]	SH (verbal) SA	Professional	Work settings (psychiatry)	Positive	Moderate	No
James et al., 2011 [62]	SH	Professional	Work experience (<10 years)	Negative	Moderate	Yes
Kobayashi et al., 2020 [65]	SV	Professional	Ward type Professional title	Unclear	Low	Yes
Maguire & Ryan, 2007 [66]	SH	Sociodemographic	Age (40–44 years)	Positive	Moderate	Yes
McKenna et al., 2003 [67]	SH (verbal) SH (physical)	Professional	Work settings (psychiatry)	Positive	Moderate	Yes
Nijman et al., 2005 [69]	SH	Sociodemographic	Gender (female) Age (<40 years)	Positive	Moderate	Yes
Schlup et al., 2021 [71]	SV (verbal) SV (physical)	Sociodemographic	Gender (male)	Negative	Low	Yes
	SV (verbal)	Professional	Work experience (<4 years)	Positive		
			Work schedule	Unclear		
	SV (physical)		Work experience	Unclear		
			Work schedule (part-time)	Negative		
	SV (verbal) SV (physical)	Experiential	History of violence (sexual)	Positive		
	SV (verbal)		History of violence (physical) Normalization of violence	Positive		
	SV (physical)		History of violence (physical) Normalization of violence	Unclear		
Shiao et al., 2010 [72]	SH (verbal) SH (physical)	Professional	Work settings (psychiatry)	Positive	Low	Yes
	SA			Unclear		
Yang et al. 2018 [73]	SH (verbal) SH (physical)	Sociodemographic	Gender (male)	Positive	Low	Yes
			Marital status Education	Unclear		
		Professional	Ward type (acute) Work shift (night shift) Work activities (routine activities) Patient gender (male)	Positive		
			Professional title Employment type Location of events Patient age Patient residence	Unclear		
		Experiential	Perception of preventability	Unclear		
Zeng et al., 2013 [75]	SA	Sociodemographic	Gender (male)	Positive	Low	Yes
	SH (verbal) SH (physical)	Sociodemographic	Gender	Unclear		
Zeng et al., 2020 [76]	SH	Professional	Work experience (shorter)	Positive	Low	Yes

Legend: SA = sexual assault; SH = sexual harassment; SV = sexual violence. Each study may contribute multiple rows when multiple risk factors or forms of SV were examined.

**Table 4:** Risk of bias in the included studies.

Cohort studies (assessed using the JBI checklist for cohort study, 11 items)														
Reference	Q1	Q2	Q3	Q4	Q5	Q6	Q7	Q8	Q9	Q10	Q11	Total	%	Risk
Chen et al., 2011 [57]	Y	Y	Y	U	U	U	Y	Y	Y	U	Y	7/11	63.63	Moderate
Chen et al., 2009 [60]	Y	Y	Y	U	U	U	U	Y	Y	U	Y	6/11	54.54	Moderate
Qualitative studies (assessed using the JBI checklist for qualitative research, 10 items)														
Reference	Q1	Q2	Q3	Q4	Q5	Q6	Q7	Q8	Q9	Q10	-	Total	%	Risk
Abu Khait et al., 2022 [54]	U	Y	Y	Y	Y	U	Y	Y	Y	Y	-	8/10	80.00	Low
Alyousef & Alhamidi, 2022 [56]	Y	Y	Y	Y	Y	U	Y	Y	Y	Y	-	9/10	90.00	Low
Yosep et al., 2023 [74]	U	Y	Y	Y	Y	U	U	Y	Y	Y	-	8/10	80.00	Low
Zwane et al., 2022 [77]	U	Y	Y	Y	Y	U	Y	Y	Y	Y	-	8/10	80.00	Low
Cross-sectional studies (assessed using the JBI checklist for analytical cross-sectional study, 8 items)														
Reference	Q1	Q2	Q3	Q4	Q5	Q6	Q7	Q8	-	-	-	Total	%	Risk
Abu El-Kass et al., 2025 [53]	Y	Y	Y	Y	U	N	Y	Y	-	-	-	6/8	75.00	Low
Alenezi, 2024 [55]	Y	Y	Y	Y	Y	Y	U	Y	-	-	-	7/8	87.50	Low
Chen et al., 2008 [58]	U	Y	Y	Y	Y	Y	U	Y	-	-	-	6/8	75.00	Low
Chen et al., 2009 [59]	Y	Y	Y	Y	U	U	U	Y	-	-	-	5/8	62.50	Moderate
Hesketh et al., 2003 [61]	Y	Y	U	Y	U	N	Y	Y	-	-	-	5/8	62.50	Moderate
James et al., 2011 [62]	Y	Y	Y	Y	U	N	U	Y	-	-	-	5/8	62.50	Moderate
Jonker et al., 2008 [63]	Y	Y	Y	Y	U	Y	Y	Y	-	-	-	7/8	87.50	Low
Khoshknab et al., 2012 [64]	Y	Y	Y	Y	U	N	U	Y	-	-	-	5/8	62.50	Moderate
Kobayashi et al., 2020 [65]	Y	Y	Y	Y	U	Y	Y	Y	-	-	-	7/8	87.50	Low
Maguire & Ryan, 2007 [66]	Y	Y	Y	Y	U	N	U	Y	-	-	-	5/8	62.50	Moderate
McKenna et al., 2003 [67]	U	Y	Y	Y	U	U	Y	Y	-	-	-	5/8	62.50	Moderate
Nijman et al., 2005 [69]	Y	Y	Y	Y	U	U	U	Y	-	-	-	5/8	62.50	Moderate
Niu et al., 2019 [70]	Y	Y	Y	Y	U	Y	U	Y	-	-	-	6/8	75.00	Low
Schlup et al., 2021 [71]	Y	Y	Y	Y	U	Y	U	Y	-	-	-	6/8	75.00	Low
Shiao et al., 2010 [72]	Y	Y	Y	Y	U	Y	Y	Y	-	-	-	7/8	87.50	Low
Yang et al. 2018 [73]	Y	Y	Y	Y	U	U	Y	Y	-	-	-	6/8	75.00	Low
Zeng et al., 2013 [75]	Y	Y	Y	Y	U	Y	Y	Y	-	-	-	7/8	87.50	Low
Zeng et al., 2020 [76]	Y	Y	Y	Y	U	Y	Y	Y	-	-	-	7/8	87.50	Low
Mixed-method studies (assessed using the MMAT checklist for mixed-method studies, 7 items)														
Reference	Q1	Q2	Q3	Q4	Q5	Q6	Q7	-	-	-	-	Total	%	Risk
Moylan and Cullian, 2011 [68]	Y	Y	U	Y	Y	Y	U					4/7	57.14	Moderate

Legend: Y = yes, N = no, U = unclear, JBI = Joanna Briggs Institute, MMAT = Mixed Methods Appraisal Tool. The level of bias risk was considered: high < 50% “yes” scores; moderate = 50–70% “yes” scores; low > 70% “yes” scores.

**Table 5:** Quality of evidence assessment using the GRADE approach.

Outcome	Scale	Mean Score (±SD)	Reference	Sample	Form of Sexual Violence	Association	Quality of Evidence (GRADE)	Explanation
QoL	SF-36	QoL overall: 93.1 (±11.5)	[53,76]	1620 MHNs	SH	Negative	⊕⊕◯◯ Low	Significant negative associations (*p* < 0.05) were found for overall QoL and its four dimensions (social, physical, psychological, and environmental) in two cross-sectional studies. No criteria emerged to justify an increase or decrease in the quality of the evidence. Caution is advised in interpretation.
	WHOQOL-BREF	QoL social: 13.1 (±3.1) QoL physical: 13.4 (±2.3) QoL psychological: 12.9 (±2.6) QoL environmental: 11.5 (±2.6)						
Burnout	MBI-GS	EE: 1.99 (±1.25) DP: 1.24 (±1.24) RPA: 2.18 (±1.21)	[73]	245 MHNs	SH	Positive	⊕⊕◯◯ Low	Significant positive associations (*p* < 0.05) were found for EE in one cross-sectional study. No criteria emerged to justify an increase or decrease in the quality of the evidence. Caution is advised in interpretation.
Day lost from work	-	1.2 days	[69]	154 MHNS	SH SA	Positive	⊕◯◯◯ Very low	Significant positive association (*p* < 0.05) was found for days lost from work in one cross-sectional study. A reduction in evidence quality is necessary due to the utilization of unstructured tools (additional items), the limited sample size, and the result of the risk of bias assessment. Interpretation should proceed with great caution.

Legend: DP = depersonalization; EE = emotional exhaustion; MBI-GS = Maslach Burnout Inventory-General Survey; MHNs = mental health nurses; QoL = quality of life; RPA = reduced personal accomplishment; SA = sexual assault; SF-36 = 36-Item Short-Form Health Survey; SH = sexual harassment; QoL = quality of life; WHOQOL-BREF = World Health Organization Quality of Life Schedule Brief. GRADE level for certainty of evidence: ⊕⊕◯◯ (Low) = The authors’ confidence in the association is limited. The true association may be substantially different from the observed one. ⊕◯◯◯ (Very Low) = The authors’ confidence in the association is very low. The true association is likely to be substantially different from the observed one.

## Data Availability

No new data were created or analyzed in this study. Data sharing does not apply to this article.

## Public Involvement Statement

No public involvement in any aspect of this research.

## Guidelines and Standards Statement

This manuscript was drafted against the Preferred Reporting Items for Systematic Reviews and Meta-Analyses (PRISMA) guidelines for systematic review.

## Abbreviations

The following abbreviations are used in this manuscript:

MHNs	Mental Health Nurses
HCWs	Healthcare Workers
MHSs	Mental Health Settings
SV	Sexual Violence
QoL	Quality of Life
PTSD	Post-Traumatic Stress Disorder
PRISMA	Preferred Reporting Items for Systematic Reviews and Meta-Analyses
PROSPERO	International Prospective Register of Systematic Reviews

## Appendix A

**Table A1:** PRISMA Checklist.

Section and Topic	Item #	Checklist Item	Location Where the Item is Reported
TITLE			
Title	1	Identify the report as a systematic review.	Pag. 1
ABSTRACT			
Abstract	2	See the PRISMA 2020 for Abstracts checklist.	Pag. 1
INTRODUCTION			
Rationale	3	Describe the rationale for the review in the context of existing knowledge.	Pag. 2–3
Objectives	4	Provide an explicit statement of the objective(s) or question(s) the review addresses.	Pag. 3
METHODS			
Eligibility criteria	5	Specify the inclusion and exclusion criteria for the review and how studies were grouped for the syntheses.	Pag. 3
Information sources	6	Specify all databases, registers, websites, organizations, reference lists, and other sources searched or consulted to identify studies. Specify the date when each source was last searched or consulted.	Pag. 3
Search strategy	7	Present the full search strategies for all databases, registers, and websites, including any filters and limits used.	Pag. 4
Selection process	8	Specify the methods used to decide whether a study met the inclusion criteria of the review, including how many reviewers screened each record and each report retrieved, whether they worked independently, and, if applicable, details of automation tools used in the process.	Pag. 4
Data collection process	9	Specify the methods used to collect data from reports, including how many reviewers collected data from each report, whether they worked independently, any processes for obtaining or confirming data from study investigators, and, if applicable, details of automation tools used in the process.	Pag. 4
Data items	10a	List and define all outcomes for which data were sought. Specify whether all results that were compatible with each outcome domain in each study were sought (e.g., for all measures, time points, analyses), and if not, the methods used to decide which results to collect.	Pag. 4
	10b	List and define all other variables for which data were sought (e.g., participant and intervention characteristics, funding sources). Describe any assumptions made about any missing or unclear information.	Pag. 4
Study risk of bias assessment	11	Specify the methods used to assess risk of bias in the included studies, including details of the tool(s) used, how many reviewers assessed each study, and whether they worked independently, and if applicable, details of automation tools used in the process.	Pag. 4–5
Effect measures	12	Specify for each outcome the effect measure(s) (e.g., risk ratio, mean difference) used in the synthesis or presentation of results.	Pag. 5
Synthesis methods	13a	Describe the processes used to decide which studies were eligible for each synthesis (e.g., tabulating the study intervention characteristics and comparing against the planned groups for each synthesis (item #5)).	Pag. 5
	13b	Describe any methods required to prepare the data for presentation or synthesis, such as handling of missing summary statistics or data conversions.	Pag. 5
	13c	Describe any methods used to tabulate or visually display the results of individual studies and syntheses.	Pag. 5
	13d	Describe any methods used to synthesize results and provide a rationale for the choice(s). If meta-analysis was performed, describe the model(s), method(s) to identify the presence and extent of statistical heterogeneity, and software package(s) used.	Pag. 5
	13e	Describe any methods used to explore possible causes of heterogeneity among study results (e.g., subgroup analysis, meta-regression).	-
	13f	Describe any sensitivity analyses conducted to assess the robustness of the synthesized results.	-
Reporting bias assessment	14	Describe any methods used to assess the risk of bias due to missing results in a synthesis (arising from reporting biases).	Pag. 4
Certainty assessment	15	Describe any methods used to assess certainty (or confidence) in the body of evidence for an outcome.	Pag. 5
RESULTS			
Study selection	16a	Describe the results of the search and selection process, from the number of records identified in the search to the number of studies included in the review, ideally using a flow diagram.	Pag. 5–6 Figure 1
	16b	Cite studies that might appear to meet the inclusion criteria, but which were excluded, and explain why they were excluded.	Pag. 5–6 Figure 1
Study characteristics	17	Cite each included study and present its characteristics.	Pag. 6–11 Table 1
Risk of bias in studies	18	Present assessments of risk of bias for each included study.	Pag. 15–16 Table 4
Results of individual studies	19	For all outcomes, present, for each study: (a) summary statistics for each group (where appropriate) and (b) an effect estimate and its precision (e.g., confidence/credible interval), ideally using structured tables or plots.	Pag. 11–15 Table 2 and Table 3
Results of syntheses	20a	For each synthesis, briefly summarize the characteristics and risk of bias among contributing studies.	Pag. 11–15 Table 2 and Table 3
	20b	Present the results of all statistical syntheses conducted. If meta-analysis was done, present for each the summary estimate and its precision (e.g., confidence/credible interval) and measures of statistical heterogeneity. If comparing groups, describe the direction of the effect.	Pag. 11–15 Table 2 and Table 3
	20c	Present the results of all investigations of possible causes of heterogeneity among study results.	Pag. 11–15 Table 2 and Table 3
	20d	Present the results of all sensitivity analyses conducted to assess the robustness of the synthesized results.	-
Reporting biases	21	Present assessments of risk of bias due to missing results (arising from reporting biases) for each synthesis assessed.	Pag. 11–15 Table 2 and Table 3
Certainty of evidence	22	Present assessments of certainty (or confidence) in the body of evidence for each outcome assessed.	Pag. 16–17 Table 5
DISCUSSION			
Discussion	23a	Provide a general interpretation of the results in the context of other evidence.	Pag. 17–18
	23b	Discuss any limitations of the evidence included in the review.	Pag. 19–20
	23c	Discuss any limitations of the review processes used.	Pag. 19–20
	23d	Discuss implications of the results for practice, policy, and future research.	Pag. 19
OTHER INFORMATION			
Registration and protocol	24a	Provide registration information for the review, including the register name and registration number, or state that the review was not registered.	Pag. 3
	24b	Indicate where the review protocol can be accessed, or state that a protocol was not prepared.	Pag. 3
	24c	Describe and explain any amendments to information provided at registration or in the protocol.	-
Support	25	Describe sources of financial or non-financial support for the review, and the role of the funders or sponsors in the review.	Pag. 20
Competing interests	26	Declare any competing interests of review authors.	Pag. 21
Availability of data, code, and other materials	27	Report which of the following are publicly available and where they can be found: template data collection forms; data extracted from included studies; data used for all analyses; analytic code; any other materials used in the review.	Pag. 21

**Table A2:** Final search strategy and results for each database.

ID	Final Search Strategy for PubMed/Medline (Search Conducted on 19 July 2025)	Results
1	((“Nurses” [Mesh]) OR (“nurses”) OR (“nurse”) OR (“Licensed Practical Nurses” [Mesh]) OR (“Nursing Assistants ”[Mesh])) OR (((“nursing”) OR (“nurse”) OR (“nurses”)) AND ((“staff”) OR (“team”) OR (“worker”) OR (“workers”) OR (“personnel”) OR (“professional”) OR (“professionals”) OR (“workforce”) OR (“assistant”) OR (“assistants”) OR (“auxiliary”) OR (“aid”) OR (“aides”) OR (“employee”) OR (“employees”) OR (“practitioner”) OR (“practitioners”)))	653,602
2	((“Psychiatric Department, Hospital” [Mesh]) OR (“Hospitals, Psychiatric” [Mesh]) OR (“acute psychiatric unit”) OR (“acute inpatient psychiatric unit”) OR (“acute mental health unit”) OR (“acute inpatient mental health unit”)) OR (((“psychiatric”) OR (“mental health”)) AND ((“department”) OR (“hospital”) OR (“unit”) OR (“ward”)))	528,993
3	(((“Nurses” [Mesh]) OR (“nurses”) OR (“nurse”) OR (“Licensed Practical Nurses” [Mesh]) OR (“Nursing Assistants” [Mesh])) OR (((“nursing”) OR (“nurse”) OR (“nurses”)) AND ((“staff”) OR (“team”) OR (“worker”) OR (“workers”) OR (“personnel”) OR (“professional”) OR (“professionals”) OR (“workforce”) OR (“assistant”) OR (“assistants”) OR (“auxiliary”) OR (“aid”) OR (“aides”) OR (“employee”) OR (“employees”) OR (“practitioner”) OR (“practitioners”)))) AND (((“Psychiatric Department, Hospital” [Mesh]) OR (“Hospitals, Psychiatric” [Mesh]) OR (“acute psychiatric unit”) OR (“acute inpatient psychiatric unit”) OR (“acute mental health unit”) OR (“acute inpatient mental health unit”)) OR (((“psychiatric”) OR (“mental health”)) AND ((“department”) OR (“hospital”) OR (“unit”) OR (“ward”))))	28,426
4	((“Sex Offenses” [Mesh]) OR (“Sexual Harassment” [Mesh]) OR (“rape”)) OR ((“sexual”) AND ((“Aggression” [Mesh]) OR (“aggression”) OR (“Violence” [Mesh]) OR (“violence”) OR (“workplace violence”) OR (“offense”) OR (“offence”) OR (“harassment”) OR (“assault”) OR (“abuse”)))	73,518
5	((((“Nurses” [Mesh]) OR (“nurses”) OR (“nurse”) OR (“Licensed Practical Nurses” [Mesh]) OR (“Nursing Assistants” [Mesh])) OR (((“nursing”) OR (“nurse”) OR (“nurses”)) AND ((“staff”) OR (“team”) OR (“worker”) OR (“workers”) OR (“personnel”) OR (“professional”) OR (“professionals”) OR (“workforce”) OR (“assistant”) OR (“assistants”) OR (“auxiliary”) OR (“aid”) OR (“aides”) OR (“employee”) OR (“employees”) OR (“practitioner”) OR (“practitioners”)))) AND (((“Psychiatric Department, Hospital” [Mesh]) OR (“Hospitals, Psychiatric” [Mesh]) OR (“acute psychiatric unit”) OR (“acute inpatient psychiatric unit”) OR (“acute mental health unit”) OR (“acute inpatient mental health unit”)) OR (((“psychiatric”) OR (“mental health”)) AND ((“department”) OR (“hospital”) OR (“unit”) OR (“ward”))))) AND (((“Sex Offenses” [Mesh]) OR (“Sexual Harassment” [Mesh]) OR (“rape”)) OR ((“sexual”) AND ((“Aggression” [Mesh]) OR (“aggression”) OR (“Violence” [Mesh]) OR (“violence”) OR (“workplace violence”) OR (“offense”) OR (“offence”) OR (“harassment”) OR (“assault”) OR (“abuse”))))	377
ID	Final search strategy for CINAHL (search conducted on 19 July 2025)	Results
1	((MH “Nurses+”) OR (MH “Correctional Nurses”) OR (MH “Addictions Nurses”) OR (MH “Psychiatric Nurses”) OR (MH “Community Mental Health Nurses”) OR (MH “Psychiatric Mental Health Clinical Nurse Specialists”) OR (MH “Psychiatric Mental Health Nurse Practitioners”) OR (MH “Community Mental Health Nursing”) OR (MH “Nurse Practitioners”) OR (MH “Registered Nurses”) OR (MH “Staff Nurses”) OR (MH “Nursing Staff, Hospital”) OR (MH “Medical Staff”) OR (MH “Medical Staff, Hospital”) OR (MH “Practical Nurses”) OR (MH “Certified Nursing Assistants”) OR (MH “First Assistants”) OR (MH “Medical Assistants”) OR (MH “Paramedics”) OR (MH “Physician Assistants”) OR (MH “Psychiatric Technicians”) OR (“MH “Health Personnel”) OR (MH “Health Personnel, Unlicensed”))	551,333
2	(((“nursing”) OR (“nurse”) OR (“nurses”)) AND ((“staff”) OR (“team”) OR (“worker”) OR (“workers”) OR (“personnel”) OR (“professional”) OR (“professionals”) OR (“workforce”) OR (“assistant”) OR (“assistants”) OR (“auxiliary”) OR (“aid”) OR (“aides”) OR (“employee”) OR (“employees”) OR (“practitioner”) OR (“practitioners”)))	733,755
3	S1 OR S2	977,164
4	((MH “Hospitals, Psychiatric”) OR (MH “Psychiatric Units”) OR (MH “Psychiatric Service”) OR (“acute psychiatric unit”) OR (“acute inpatient psychiatric unit”) OR (“acute mental health unit”) OR (“acute inpatient mental health unit”))	24,556
5	(((“psychiatric”) OR (“mental health”)) AND ((“department”) OR (“hospital”) OR (“unit”) OR (“ward”) OR (“service”)))	116,643
6	S4 OR S5	127,183
7	((MH “Sexual Harassment”) OR (MH “Sexual Abuse”) OR (MH “Rape”) OR (“rape”))	36,617
8	((“sexual”) AND ((“aggression”) OR (“violence”) OR (“offense”) OR (“offence”) OR (“harassment”) OR (“assault”) OR (“abuse”)))	77,543
9	S7 OR S8	81,929
10	S3 AND S6 AND S9	130
ID	Final search strategy for SCOPUS (search conducted on 19 July 2025)	Results
1	TITLE-ABS-KEY (((“nurses”) OR (“nurse”) OR (“paramedics”)))	542,470
2	TITLE-ABS-KEY ((((“nursing”) OR (“nurse”) OR (“nurses”)) AND ((“staff”) OR (“team”) OR (“worker”) OR (“workers”) OR (“personnel”) OR (“professional”) OR (“professionals”) OR (“workforce”) OR (“assistant”) OR (“assistants”) OR (“auxiliary”) OR (“aid”) OR (“aides”) OR (“employee”) OR (“employees”) OR (“practitioner”) OR (“practitioners”))))	442,194
3	(TITLE-ABS-KEY (((“nurses”) OR (“nurse”) OR (“paramedics”)))) OR (TITLE-ABS-KEY ((((“nursing”) OR (“nurse”) OR (“nurses”)) AND ((“staff”) OR (“team”) OR (“worker”) OR (“workers”) OR (“personnel”) OR (“professional”) OR (“professionals”) OR (“workforce”) OR (“assistant”) OR (“assistants”) OR (“auxiliary”) OR (“aid”) OR (“aides”) OR (“employee”) OR (“employees”) OR (“practitioner”) OR (“practitioners”)))))	691,345
4	TITLE-ABS-KEY (((“acute psychiatric unit”) OR (“acute inpatient psychiatric unit”) OR (“acute mental health unit”) OR (“acute inpatient mental health unit”)))	473
5	TITLE-ABS-KEY ((((“psychiatric”) OR (“mental health”)) AND ((“department”) OR (“hospital”) OR (“unit”) OR (“ward”) OR (“service”))))	322,367
6	(TITLE-ABS-KEY (((“acute psychiatric unit”) OR (“acute inpatient psychiatric unit”) OR (“acute mental health unit”) OR (“acute inpatient mental health unit”))))) OR (TITLE-ABS-KEY ((((“psychiatric”) OR (“mental health”)) AND ((“department”) OR (“hospital”) OR (“unit”) OR (“ward”) OR (“service”)))))	322,367
7	TITLE-ABS-KEY (((“sex offenses”) OR (“rape”)))	49,874
8	TITLE-ABS-KEY (((“sexual”) AND ((“aggression”) OR (“violence”) OR (“offense”) OR (“offence”) OR (“harassment”) OR (“assault”) OR (“abuse”))))	131,273
9	(TITLE-ABS-KEY (((“sex offenses”) OR (“rape”)))) OR (TITLE-ABS-KEY (((“sexual”) AND ((“aggression”) OR (“violence”) OR (“offense”) OR (“offence”) OR (“harassment”) OR (“assault”) OR (“abuse”))))	158,704
10	((TITLE-ABS-KEY (((“nurses”) OR (“nurse”) OR (“paramedics”)))) OR (TITLE-ABS-KEY ((((“nursing”) OR (“nurse”) OR (“nurses”)) AND ((“staff”) OR (“team”) OR (“worker”) OR (“workers”) OR (“personnel”) OR (“professional”) OR (“professionals”) OR (“workforce”) OR (“assistant”) OR (“assistants”) OR (“auxiliary”) OR (“aid”) OR (“aides”) OR (“employee”) OR (“employees”) OR (“practitioner”) OR (“practitioners”)))))) AND ((TITLE-ABS-KEY (((“acute psychiatric unit”) OR (“acute inpatient psychiatric unit”) OR (“acute mental health unit”) OR (“acute inpatient mental health unit”)))) OR (TITLE-ABS-KEY ((((“psychiatric”) OR (“mental health”)) AND ((“department”) OR (“hospital”) OR (“unit”) OR (“ward”) OR (“service”)))))) AND ((TITLE-ABS-KEY (((“sex offenses”) OR (“rape”)))) OR (TITLE-ABS-KEY (((“sexual”) AND ((“aggression”) OR (“violence”) OR (“offense”) OR (“offence”) OR (“harassment”) OR (“assault”) OR (“abuse”))))))	385
ID	Final search strategy for Web of Science (search conducted on 19 July 2025)	Results
1	ALL = (((“nurses”) OR (“nurse”) OR (“paramedics”)))	334,067
2	ALL = ((((“nursing”) OR (“nurse”) OR (“nurses”)) AND ((“staff”) OR (“team”) OR (“worker”) OR (“workers”) OR (“personnel”) OR (“professional”) OR (“professionals”) OR (“workforce”) OR (“assistant”) OR (“assistants”) OR (“auxiliary”) OR (“aid”) OR (“aides”) OR (“employee”) OR (“employees”) OR (“practitioner”) OR (“practitioners”))))	288,812
3	#1 OR #2	461,983
4	ALL = (((“acute psychiatric unit”) OR (“acute inpatient psychiatric unit”) OR (“acute mental health unit”) OR (“acute inpatient mental health unit”)))	258
5	ALL = ((((“psychiatric”) OR (“mental health”)) AND ((“department”) OR (“hospital”) OR (“unit”) OR (“ward”) OR (“service”))))	361,107
6	#4 OR #5	361,107
7	ALL = (((“sex offenses”) OR (“rape”)))	33,392
8	ALL = (((“sexual”) AND ((“aggression”) OR (“violence”) OR (“offense”) OR (“offence”) OR (“harassment”) OR (“assault”) OR (“abuse”))))	95,970
9	#7 OR #8	118,843
10	#3 AND #6 AND #9	493
ID	Final search strategy for PsycInfo (search conducted on 19 July 2025)	Results
1	((DE “Nurses”) OR (DE “Nurse Practitioners”) OR (DE “Psychiatric Nurses”) OR (DE “Psychiatric Hospital Staff”) OR (DE “Mental Health Personnel”) OR (DE “Allied Health Personnel”) OR (DE “Health Personnel”) OR (DE “Medical Personnel”) OR (DE “Physician Assistants”) OR (DE “Direct Care Workers”) OR (DE “Paramedics”))	94,696
2	(((“nursing”) OR (“nurse”) OR (“nurses”)) AND ((“staff”) OR (“team”) OR (“worker”) OR (“workers”) OR (“personnel”) OR (“professional”) OR (“professionals”) OR (“workforce”) OR (“assistant”) OR (“assistants”) OR (“auxiliary”) OR (“aid”) OR (“aides”) OR (“employee”) OR (“employees”) OR (“practitioner”) OR (“practitioners”)))	108,788
3	S1 OR S2	156,643
4	((DE “Psychiatric Hospitals”) OR (DE “Psychiatric Units”) OR (DE “Psychiatric Hospitalization”) OR (DE “Psychiatric Clinics”) OR (DE “Sanatoriums”) OR (DE “Mental Health Services”) OR (“acute psychiatric unit”) OR (“acute inpatient psychiatric unit”) OR (“acute mental health unit”) OR (“acute inpatient mental health unit”))	71,302
5	(((“psychiatric”) OR (“mental health”)) AND ((“department”) OR (“hospital”) OR (“unit”) OR (“ward”) OR (“service”)))	512,368
6	S4 OR S5	533,023
7	((DE “Sexual Violence”) OR (DE “Sex Offenses”) OR (DE “Sexual Harassment”) OR (DE “Sexual Abuse”) OR (DE “Sexual Coercion”) OR (DE “Rape”) OR (“rape”))	50,728
8	((“sexual”) AND ((“aggression”) OR (“violence”) OR (“offense”) OR (“offence”) OR (“harassment”) OR (“assault”) OR (“abuse”)))	80,726
9	S7 OR S8	89,747
10	S3 AND S6 AND S9	636
